# Capsule Endoscopy for Ileitis with Potential Involvement of Other Sections of the Small Bowel

**DOI:** 10.1155/2016/9804783

**Published:** 2016-01-12

**Authors:** Hyun Seok Lee, Yun Jeong Lim

**Affiliations:** ^1^Department of Internal Medicine, Kyungpook National University School of Medicine, Colon Cancer Center, Kyungpook National University Medical Center, 807 Hoguk-ro, Buk-gu, Daegu 41404, Republic of Korea; ^2^Department of Internal Medicine, Dongguk University, College of Medicine, Dongguk University Ilsan Hospital, 27 Dongguk-ro, Ilsandong-gu, Goyang 10326, Republic of Korea

## Abstract

Ileitis is defined as inflammation of the ileum. This condition includes ulcers, aphthous ulcers, erosions, and nodular or erythematous mucosa. Various etiologies are associated with ileitis. Crohn's disease, ulcerative colitis, medications such as nonsteroidal anti-inflammatory drugs, infectious conditions, neoplasms, infiltrative disorders, vasculitides, spondyloarthritis, endometriosis, and radiation therapy-related conditions involve the ileum. However, the differential diagnosis of terminal ileitis can be difficult in many cases. Video capsule endoscopy (VCE) has become a useful tool for the diagnosis of a variety of small bowel lesions. This review describes each of the various conditions associated with ileitis and the diagnostic value of VCE for ileitis, which may help identify and evaluate these conditions in clinical practice. Based on the information provided by VCE, a definitive diagnosis could be made using the patients' medical history, clinical course, laboratory and ileocolonoscopic findings, radiologic imaging findings, and histologic findings.

## 1. Introduction

Intubation and observation of the terminal ileum have become a standard procedure during routine screening colonoscopy, as well as in the evaluation and management of patients suspected or known to have lesions of the small bowel, including the ileum. During ileocolonoscopy, some individuals may present with various mucosal lesions of the terminal ileum. These may be limited only to the terminal ileum or manifest as part of other small bowel lesions [1, 2].

**Figure 1:**
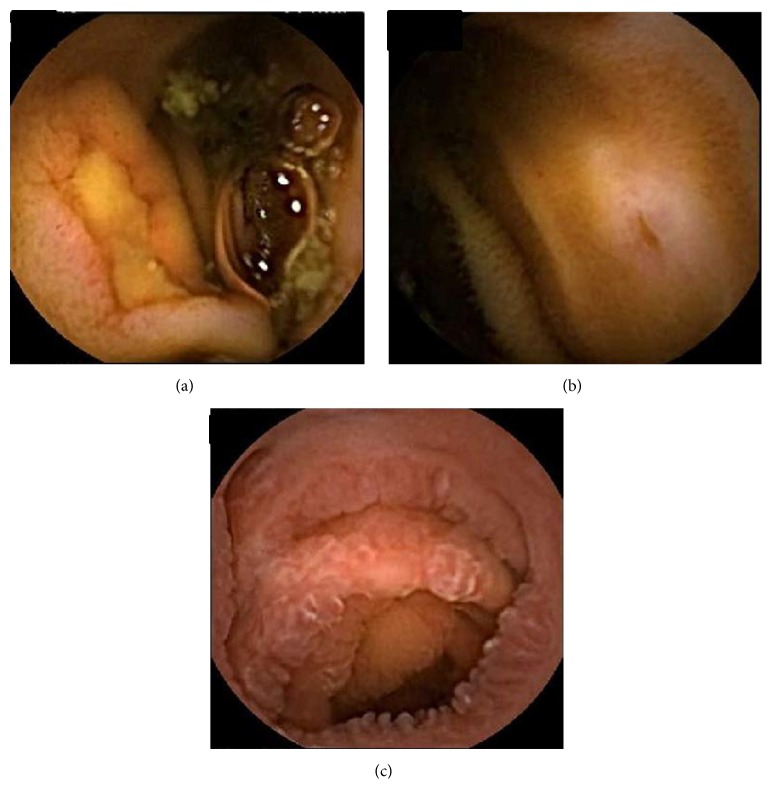
(a–c) Video capsule endoscopy findings for ileitis: (a) ulcer, (b) erosion, and (c) edematous mucosa.

Ileitis is defined as inflammation of the ileum [3] and includes ulcers, aphthous ulcers, erosions, and nodular lesions and edematous or erythematous mucosa (Figure 1) [4, 5]. Crohn's disease (CD) affects any part of the gastrointestinal tract, and involvement of the terminal ileum is frequent [1]. Many studies on ileitis have focused on CD. However, CD does not cause all cases of ileitis. Multiple other etiologies are associated with ileitis. These include various infections, vasculitis, spondyloarthritis, and drug-related factors such as nonsteroidal anti-inflammatory drugs (NSAIDs) (Table 1). The diagnosis of the cause of ileitis is important because patients require appropriate treatment for their condition, and misdiagnosis may delay patient management and worsen their condition [3, 5].

Video capsule endoscopy (VCE) has become a useful tool for the diagnosis of a variety of small bowel lesions. VCE is a noninvasive method for complete visualization and assessment of the mucosal surface. It is a safe technique without any reported mortalities. One of the risks associated with VCE is retention of the capsule. Patients with suspected CD have approximately 1 percent retention rate [6]. Careful consideration is necessary before performing VCE on any patient with the potential for capsule retention. Cases of capsule retention can often be managed conservatively, resulting in spontaneous passage of the capsule. If the capsule does not pass spontaneously after conservative medical therapy, it may be retrieved by device-assisted enteroscopy (DAE). Although conservative approaches or attempts at endoscopic capsule retrieval are unsuccessful in some cases, only a minority of patients will need to undergo surgery in order to retrieve a retained capsule. This surgical intervention not only removes the capsule but also could be used for the treatment of the underlying cause of the retention, such as a stricture or tumor [7–10].

The clinical implications of these ileal lesions that were identified during ileocolonoscopy and the guidelines for their management remain uncertain. This review describes each of the various conditions associated with ileitis, which may have involved only the ileum or other small bowel lesions (Table 1), and the role of VCE for ileitis, which may help identify and evaluate these conditions in clinical practice.

## 2. Differential Diagnoses and the Role of the VCE in Patients with Ileitis in Clinical Practice

### 2.1. Inflammatory Bowel Disease

VCE has been widely used for the diagnosis and monitoring of patients with inflammatory bowel disease (IBD), principally CD. About 30% of the patients with CD have exclusive small bowel involvement [11], and their diagnosis is often missed if the decision is based solely on ileocolonoscopic findings. VCE is now considered an important technique for monitoring small bowel CD and has also been employed in the management of patients with unclassified IBD [12]. A previous prospective study evaluated the diagnostic accuracies of VCE, magnetic resonance enterography (MRE), and computed tomography enterography (CTE), in 93 patients with suspected or newly diagnosed CD compared to that of ileocolonoscopy [13]. The sensitivities and specificities for the diagnosis of CD in the terminal ileum were 100% and 91% by VCE, 81% and 86% with MRE, and 76% and 85% for CTE, respectively. VCE is therefore more accurate for diagnosing subtle small bowel lesions than any other modality. VCE could be the first-line modality for the detection of ileal CD that is beyond the reach of colonoscopy [12, 13].

IBD cannot be classified as CD or ulcerative colitis (UC) using ileocolonoscopy and pathologic criteria in 10–15% of the patients. At least 30% of these patients with unclassified IBD will be reclassified as having CD during the course of their diseases, usually after identification of small bowel lesions [14, 15]. Several studies have evaluated the utility of VCE for reclassification of patients with unclassified IBD. A study on the diagnostic yield of VCE in patients with UC or unclassified IBD was conducted on 120 individuals. Overall, 19 out of 120 patients (15.8%) had VCE findings consistent with the diagnosis of CD. Among these 19 patients with positive findings on VCE, 18 had also previously undergone a small-bowel follow-through study and only one showed findings consistent with CD [16]. Another multicentric study evaluated the value of VCE in increasing diagnostic accuracy in patients with unclassified IBD. Thirty patients with unclassified IBD were included in the study. Among them, 5 were diagnosed with CD. However, interestingly, CD was diagnosed on repeated ileocolonoscopy with biopsies, in 6 out of 25 VCE-negative patients. VCE is a potentially clinically useful tool for categorizing patients with unclassified IBD [17].

Backwash ileitis refers to inflammation of the terminal ileum in patients with UC. Although the term “backwash” indicates exposure of the mucosa to the reflux of cecal contents, the precise pathogenesis is not well understood [3]. The mucosal inflammation patterns of the cecum, ascending colon, and terminal ileum are often similar to one another. The severity of ileal inflammation paralleled the severity of colonic inflammation and was more common in patients with pancolitis and cecal involvement compared to those with left-sided colitis [18, 19]. Backwash ileitis can be distinguished endoscopically from the ileitis observed in CD, on the basis of the absence of distinct ulcers in the terminal ileum [20]. Mucosal biopsies may help distinguish CD from UC in patients with backwash ileitis. The presence of granulomatous inflammation on histology also indicates ileitis in CD patients [3]. However, in the absence of granulomata or indistinguishable ileitis in IBD patients, VCE may be useful. Although VCE may not play a significant additional role in the diagnosis or management of backwash ileitis itself, as identified by ileocolonoscopy, it may provide useful information for reclassifying unclassified IBD with ileitis or altering the indeterminate current diagnosis (UC or CD). In a retrospective cohort analysis of patients with previously diagnosed IBD, 4 out of 5 patients with IC and 1 of 2 patients with unclassified IBD had their disease reclassified to CD based on newly diagnosed small bowel mucosal lesions [21].

### 2.2. Drug-Related Ileitis

Most drugs may cause a diffuse small bowel lesion that includes the ileum. Ulcerations due to NSAIDs can occur in the stomach and duodenum. The small bowel and colon are also susceptible to the adverse effects of NSAIDs [22]. NSAID enteropathy is usually subclinical, although some patients may present with various NSAIDs-induced injuries, such as ulcers, erosions, strictures, and perforations in the small bowel, including the ileum [23]. The pathogenesis is believed to involve the inhibition of intestinal prostaglandin synthesis [24].

Diagnosis may be made using direct visualization methods, such as VCE, ileocolonoscopy, and DAE. NSAID enteropathy, including ileitis, is suspected in patients with a history of NSAID use. Both elderly patients and those taking long-term NSAIDs tend to be at higher risk of NSAID enteropathy [24]. Endoscopic and VCE findings in these patients include ulcerations, erosions, and strictures. These symptoms or endoscopic findings should improve following the withdrawal of NSAIDs [3].

NSAIDs frequently lead to ileitis or colitis resembling CD. They also exacerbate preexisting CD. NSAID-induced enteropathy is often misdiagnosed as CD because of the pathological similarity between these two diseases. Since CD usually causes long, thick inflammatory strictures rather than thin, fibrotic diaphragms and presents with ulcers that are often deeper, longitudinal, and more irregular than the sharply demarcated lesions of NSAID enteropathy, the two conditions can be distinguished from each other on this basis. Furthermore, a cobblestone appearance, inflammatory polyps, and histologic findings of granulomas, crypt abscesses, or crypt distortion suggest CD instead of NSAID enteropathy [3, 5].

Small bowel diaphragm disease is a relatively recent clinical entity, which presents with short, symmetric ileal strictures and focal bowel wall thickening. The most common cause is the long-term use of NSAIDs, which inhibit cyclooxygenase-1. Cyclooxygenase-1 inhibition results in reduced microcirculatory blood flow, localized ischemia, and ulcers. The strictures and mucosal diaphragms developed from circumferential mucosal ulceration with subsequent contractions of scar tissue rings [25]. A recent VCE study identified mucosal diaphragms in 2% of 120 patients taking long-term NSAIDs [26]. Other conditions that can result in mucosal diaphragms include potassium intake, celiac disease, eosinophilic gastroenteritis, and radiation injury [27]. Pathologically, diaphragms appear as a disk of tissue protruding circumferentially into the intestinal lumen, reducing the lumen to a small diameter. The most common presenting symptoms are abdominal pain and anemia [25, 28].

NSAID-induced small bowel injury has been retrospectively assessed using a small bowel VCE database registry [29]. The lesions were located in the jejunum (52.8%) and ileum (27.9%) in 140 patients. The most prominent findings after performing VCE were multiple ulcerations (58.6%) and erosions or aphthous ulcers (22.9%) [29].

Several other drugs rarely cause inflammation that is confined to the ileum. Localized ulceration, fibrosis, and stenosis of the ileum with obstruction occurred in patients who ingested tablets with a combination of enteric-coated potassium chloride and hydrochlorothiazide [30]. Parenteral gold therapy is associated with inflammation with edema and ulceration that is limited to the ileum. This rare complication can develop after being treated with gold therapy for rheumatoid arthritis [31]. Other types of drug-related ileitis without diffuse small bowel lesions are associated with the use of oral contraceptives and digoxin [3, 32, 33].

### 2.3. Ileitis of Uncertain Clinical Significance

Some asymptomatic individuals may present with ileitis, such as aphthous ulcers or small ulcerations in the terminal ileum, which are unaccompanied by lesions in the ileocecal valve or colon [2]. Advances in VCE have increased the detection of small bowel lesions in healthy, asymptomatic individuals, as well as in patients with small bowel disease [2]. Some studies on VCE have reported that small bowel mucosal breaks are found in 5% to 10% of healthy individuals [34–36], although other studies have failed to detect these lesions in healthy subjects [2, 37, 38].

These isolated terminal ileal ulcerations may be one of the earlier manifestations of serious diseases, such as CD and intestinal tuberculosis. Some patients with aphthous ulcer-type CD later develop typical ulcer-type CD. In these patients, progression from aphthous ulcer lesions to overt CD requires a relatively long period. In other patients, aphthous ulcer lesions disappear or remain unchanged on follow-up visits [2, 39]. A recent study reported that CD is unlikely to develop in asymptomatic individuals with isolated ileitis [40].

### 2.4. Nodular Lymphoid Hyperplasia

Nodular lymphoid hyperplasia (NLH) of the ileum may cause ileitis. NLH of the ileum is a benign reactive process, which is also known as terminal lymphoid ileitis. It can present as an asymptomatic disease in most patients and is a rare condition in adults. Published literature on NLH mainly includes case reports and a small series of patients [1, 41, 42]. A previous study reported the case of a 13-year-old boy with a stricture of the ileum that was diagnosed as CD on small bowel follow-through. The lesion did not resolve on steroid therapy, and the patient required surgical resection of the terminal ileum. Histopathology of this region showed focal lymphoid hyperplasia instead of CD [43].

NLH of the gastrointestinal tract is characterized by the presence of multiple small nodules, which are normally between 2 and 10 mm in diameter. NLH is more commonly distributed in the small bowel and mainly involves the terminal ileum, although it may also be found in the stomach, colon, or rectum [44]. The pathogenesis is largely unknown. NLH can occur in all age groups, but it is primarily diagnosed in children and can also affect adults with or without immunodeficiency. Some patients have associated diseases, such as common variable immunodeficiency disease, selective IgA deficiency,* Giardia lamblia* infection, human immunodeficiency virus (HIV) infection, celiac disease, or* Helicobacter pylori* infection [41]. NLH in a defunctionalized colon was reported in an adult immunocompetent patient who underwent ileostomy because of localized regional ileitis [45].

A diagnosis of NLH is established by endoscopy, VCE, or small bowel barium studies and confirmed histologically. The condition is defined histologically by markedly hyperplasic, mitotically active germinal centers, and well-defined lymphocyte mantles found in the lamina propria or in the superficial submucosa [46]. A retrospective study was conducted to test the ability of MR enterography to differentiate NLH of the ileum from CD. NLH altered both subjective and quantitative MRI parameters, including the T2 signal, contrast enhancement, and mural thickness. NLH was erroneously diagnosed as CD in a blinded assessment, among four out of nine cases (44%), whereas all cases of CD were correctly classified. NLH of the ileum may be indistinguishable from CD on MR enterography [47]. When NLH is involved in the small intestine, VCE is important for the diagnosis, in order to exclude complications such as lymphoma and to determine the extent of the disease in the small bowel [41]. Treatment is directed towards associated conditions because the disorder itself generally requires no intervention. NLH is a risk factor for both intestinal and very rarely extraintestinal lymphoma. Since there is a risk of malignant transformation, surveillance VCE and small bowel series are recommended by some authors, in patients with small bowel involvement of the NLH [41, 48].

### 2.5. Ileitis Caused by Intestinal Infection

Intestinal infections usually present with an acute episode of diarrhea, which resolves spontaneously. As a result, an endoscopic assessment is not needed in most patients. In contrast to acute diarrhea, patients with persistent or chronic diarrhea require further evaluation. Endoscopy is the main diagnostic procedure as it facilitates the examination of the mucosa. Biopsy specimens can also be obtained in order to identify the causal pathogens. Biopsies are frequently performed during ileocolonoscopy. These procedures have a limited range of examination because a large part of the small intestines is excluded. As a result, VCE is an extremely useful tool, since it allows assessments of the intestinal mucosa with a high diagnostic yield and can have a direct impact on the management of such patients. VCE can detect erosions or ulcers involving the intestinal mucosa in patients with a variety of bacterial infections, viruses, fungus, or parasites (Table 1). Moreover, the lesions detected by VCE can be subsequently confirmed by biopsies obtained during DAE [49].

Although intestinal infections by many pathogens can involve the ileum,* Yersinia* and abdominal tuberculosis infections may specifically involve the ileum. The ileocecal region is the most common site of intestinal tuberculosis caused by a* Mycobacterium tuberculosis* infection [50]. Ileocolonoscopic findings of intestinal tuberculosis may include ulcers, strictures, pseudopolyps, nodules, fistulas, or deformed ileocecal valves. The main differential diagnosis of ileocecal tuberculosis at endoscopy is CD [3, 51]. Biopsy for culture and histopathological evaluation can be useful in definitively distinguishing between these two disorders. Longitudinal ulcers, skip lesions, anorectal lesions, aphthous ulcers, and a cobblestone appearance were significantly more frequent in patients with CD than in those with intestinal tuberculosis. Transverse ulcers, a patulous ileocecal valve, scars or pseudopolyps, and the involvement of fewer than four segments were more commonly observed in patients with intestinal tuberculosis than in those with CD [52].

*Yersinia enterocolitica *infection occurs mainly in the terminal ileum and ileocecal valve and causes mucosal ulceration and thickening of the ileal wall. The diagnosis is made most directly by ileocolonoscopy with biopsy and culture. Endoscopic features of* Yersinia* infection include round or oval elevations with ulcerations in the terminal ileum, and small ulcers may be detected on the ileocecal valve and the cecum. In contrast to CD ulcers,* Yersinia* ulcers are mostly uniform in size and shape [1, 53].

Although VCE can detect erosions or ulcers involving the intestinal mucosa in patients with an intestinal infection, VCE may not have a significant role in the diagnosis and management of these conditions because they usually are treated with empirical agents and supportive measures in actual clinical practice.

### 2.6. Neoplasms

Ileitis presents with ulcers, erosions, nodular lesions, and edematous or erythematous mucosa of the ileum. Small bowel neoplasms may also manifest as the appearance of ileitis when the small bowel lesions include the ileum or only involve the ileum. Small bowel malignancy may represent less than 2% of all malignant tumors of the gastrointestinal tract. VCE enables a more detailed inspection of the small bowel. A registry-based series of 67,843 patients with small bowel tumors was reported to the National Cancer Database. In these patients, the distribution of primary tumors in the small intestine included carcinoid tumors (45%), lymphomas (21%), adenocarcinomas (13%), and sarcomas of the ileum (15%) [54]. These lesions usually manifest as wall thickenings, areas of luminal narrowing, and small ulcers and erosions that resemble ileitis.

The utility of VCE in the diagnosis of small bowel neoplasm was demonstrated in a retrospective analysis of the records of 562 patients who underwent VCE for a variety of indications, including obscure gastrointestinal bleeding and persistent abdominal pain [55]. Fifty patients (8.9%) were diagnosed with small bowel tumors, and 48% of these were malignant lesions. The types of tumors diagnosed by VCE included 8 adenocarcinomas (1.4%), 10 carcinoids (1.8%), 4 gastrointestinal stromal tumors (0.7%), 5 lymphomas (0.9%), and 3 inflammatory polyps as well as one of each of lymphangioma, lymphangiectasia, hemangioma, hamartoma, and tubular adenoma. This incidence of small bowel tumors suggests an important role for VCE in the diagnosis of patients with suspected small bowel lesions. VCE may lead to the earlier detection and treatment of small bowel tumors, including those with ileum involvement [55].

### 2.7. Infiltrative Disorders

Ileitis includes ulcers, erosions, and nodular lesions, as well as edematous or erythematous mucosa of the ileum. Infiltrative disorders may also manifest as ileitis when the small bowel lesions involve the ileum.

#### 2.7.1. Eosinophilic Enteritis

The entire gastrointestinal tract from the esophagus to the colon can be affected in patients with eosinophilic gastroenteritis, an inflammatory disorder characterized by eosinophilic infiltration of the gastrointestinal tract. Endoscopic findings of eosinophilic ileitis include erythema, polypoid lesions, erosion, or ulceration. The diagnosis is established by the presence of an elevated number of expected eosinophils on microscopic examination of biopsies of the ileal mucosa [56]. Although the cutoff value for the definition of a pathological infiltration of eosinophils is still debated, the threshold of 20 eosinophils per high power field (×400) is used for the diagnosis of eosinophilic enteritis [57, 58].

#### 2.7.2. Amyloidosis

Amyloidosis refers to the extracellular tissue deposition of fibrils composed of low molecular weight subunits of a variety of proteins. Amyloid deposition in the gastrointestinal tract is greatest in the small intestine. Patients with gastrointestinal amyloidosis usually present with bleeding, malabsorption, protein-losing gastroenteropathy, or gastrointestinal dysmotility [59]. Endoscopic findings in the ileum in these patients include erosions, ulcerations, friability, and wall thickening. The diagnosis of gastrointestinal amyloid requires a tissue biopsy with positive staining of the amyloid with Congo red or the presence of amyloid fibrils on electron microscopy [60].

#### 2.7.3. Sarcoidosis

Sarcoidosis is a systemic granulomatous disease of unknown etiology, which is characterized by the formation of noncaseating granulomas. Although the stomach is the most commonly involved portion of the gastrointestinal tract, sarcoidosis has also been described in the esophagus, small intestine, appendix, colon, rectum, pancreas, and peritoneum [61]. Endoscopy or VCE may show nodules or aphthous erosions in the small bowel, including the ileum. The diagnosis of ileal sarcoidosis is based on the presence of noncaseating granulomas in a biopsy of the affected lesion [62].

### 2.8. Vasculitides

Vasculitides may manifest as ileitis when small bowel lesions involve the ileum and are defined by the presence of inflammatory leukocytes in vessel walls with reactive damage to the mural structures. Vasculitides involving the gastrointestinal tract are usually part of a systemic process and only rarely cause ileitis. The most common vasculitides with gastrointestinal involvement include Behcet's disease, systemic lupus erythematosus, and Henoch-Schonlein purpura [3]. Discrete ulcerations are most often seen in the terminal ileum, cecum, ascending colon, and esophagus in patients with Behcet's disease [63].

Endoscopy should be performed with caution in patients with suspected gastrointestinal involvement of vasculitis because of the increased risk of perforation of the edematous, ischemic bowel. VCE may be a useful tool for the diagnosis of ileal vasculitis because noninvasive visualization of small bowel mucosal lesions, such as irregular erosions and ulcerations, is possible [64].

### 2.9. Spondyloarthritis

Ileitis has been observed in association with various features of spondyloarthritis. The clinical features of spondyloarthritis are inflammation of the axial joints, asymmetric oligoarthritis, dactylitis, and enthesitis. Spondyloarthritis includes ankylosing spondylitis, nonradiographic axial spondyloarthritis, undifferentiated spondyloarthritis, reactive arthritis (Reiter syndrome), spondyloarthritis associated with psoriasis or psoriatic arthritis, and spondyloarthritis associated with CD and UC [65]. Additional features of spondyloarthritis include bowel inflammation. There is a strong relationship between active peripheral arthritis and histological gut inflammation. Up to two-thirds of patients with spondyloarthritis have histologic signs of bowel inflammation. Two types of lesions have been identified. An acute lesion, which resembles acute bacterial ileitis and chronic ileitis, is often indistinguishable from CD [66]. Spondyloarthritis was diagnosed in 36% of patients with IBD [67]. VCE enables visualization of small bowel lesions consistent with CD in 33% of these patients [12].

### 2.10. Endometriosis

Ileal involvement of endometriosis is rarely associated with ileitis. Endometriosis is the presence of endometrial glands and stroma at extrauterine sites. Bowel endometriosis is most commonly found on the rectosigmoid colon. In a case series of 168 patients who underwent surgical treatment of endometriosis of the bowel, the terminal ileum was involved in 1% [68]. Ileal endometriosis may present with diarrhea, constipation, abdominal pain, or bloating, which may mimic CD. Although abdominal imaging techniques can identify some bowel lesions, they are unable to differentiate between endometriosis and other conditions. These lesions can be more reliably detected and evaluated via laparoscopy [68, 69].

### 2.11. Radiation Enteritis

Injury to the intestine can occur following radiation therapy for a malignant lesion and may affect the ileum, sigmoid colon, and rectum in the radiation field. Radiation enteritis tends to involve specific areas depending on the radiation ports. Chronic radiation injury is characterized by telangiectasia, a plethora of neovascularity, and spiraled vessels with ulcerated epithelium. The diagnosis is usually established by suggestive radiologic findings in patients with compatible clinical features who have a history of radiation exposure. Abdominal CT may show nonspecific thickening or stricture of bowel segments. Deep insertion into the ileum with retrograde DAE may be disturbed by abdominal adhesion. VCE can be used to identify these lesions, despite their diminutive size. A case of a patient with radiation enteritis was reported in 2007. In that patient, colonoscopy revealed normal colonic mucosa and blood passing through the ileocecal valve, but the colonoscope could not be passed into the terminal ileum. VCE indicated that the bleeding was from edematous, fissured mucosa in the ileum [70]. Another study reported the case of a middle-aged woman who received radiotherapy after surgical resection of a uterine leiomyosarcoma. She presented with severe anemia, loose stools, and abdominal pain. Abdominal CT was normal and colonoscopy revealed fresh blood and small clots on normal mucosal in the colon. The VCE demonstrated mucosal atrophy, villous edema, and stricture, as well as diffuse bleeding from the terminal ileum, and radiation enteritis was diagnosed [71]. The largest study, which involved 15 patients, concluded that VCE can safely and effectively diagnose small intestinal radiation enteritis. No episodes of capsule retention were identified [72]. In a recent case series of three patients who were treated with pelvic radiation therapy, abdominal CT and enteroclysis did not show stenosis of the small bowel and the bleeding point remained unknown. DAE could not reach the causal lesion due to the pelvic adhesion. They subsequently underwent VCE, which revealed diffuse ileitis with multiple angioectasias. Active bleeding from radiation enteritis was diagnosed with VCE without retention in each of three patients [73]. A thick wall or strictured segment of the ileum may suggest possible VCE retention, but the risk of retention is sometimes difficult to predict. If radiation enteritis is suspected as the cause of small bowel bleeding and DAE may not accomplish deeper insertion into the ileum, VCE is recommended as the initial tool for diagnosing radiation enteritis when small bowel stenosis has not been previously detected. Most of all, the risk of retention should be assessed using abdominal CT or enteroclysis before performing VCE [73].

## 3. Conclusions

Various conditions are associated with ileitis. The utility of VCE has been established in the diagnosis of CD. VCE may provide important clinical information for patients with many other conditions that cause ileitis because of its excellent visualization of the entire small bowel mucosa, including the ileum, its excellent tolerability, and safety profile. One limitation of the VCE is its lack of the tissue sampling ability. VCE has the advantage directing DAE to identify the correct location in order to obtain biopsies. The differential diagnosis of ileitis can be difficult in many cases. Based on the information provided by VCE, a definitive diagnosis can often be made using the patient's medical history, clinical course, laboratory and ileocolonoscopic findings, radiologic imaging results, and histologic findings.

## Figures and Tables

**Table 1:** Causes of ileitis based on ileocolonoscopic findings.

Inflammatory bowel disease	
Crohn's disease	
Backwash ileitis in ulcerative colitis	
Drug-related ileitis	
Nonsteroidal anti-inflammatory drug enteropathy	
Other drugs	
Ileitis of uncertain clinical significance	
Nodular lymphoid hyperplasia	
Infection	
*Actinomyces israelii*	
*Anisakis* spp.	
*Clostridium difficile*	
*Cytomegalovirus*	
*Histoplasma capsulatum*	
*Mycobacterium avium *complex	
*Mycobacterium tuberculosis*	
*Salmonella* spp.	
*Yersinia* spp.	
Neoplasms	
Lymphoma	
Adenocarcinoma	
Leiomyosarcoma	
Carcinoid tumor	
Metastatic cancer	
Infiltrative disorders	
Eosinophilic enteritis	
Amyloidosis	
Sarcoidosis	
Vasculitides	
Behcet's disease	
Systemic lupus erythematosus	
Henoch-Schonlein purpura	
Other vasculitides	
Spondyloarthritis	
Ankylosing spondylitis	
Nonradiographic axial spondyloarthritis	
Undifferentiated spondyloarthritis	
Reactive arthritis (Reiter syndrome)	
Spondyloarthritis associated with psoriasis or psoriatic arthritis	
Endometriosis	
Radiation enteritis	
